# Effect of Oxide Systems on Purity of Tool Steels Fabricated by Electro Slag Remelting

**DOI:** 10.3390/molecules30061284

**Published:** 2025-03-13

**Authors:** Josef Walek, Lenka Kunčická

**Affiliations:** 1 Department of Metallurgical Technologies, Faculty of Materials Science and Technology, VŠB—Technical University of Ostrava, 17. Listopadu 2172/15, 708 00 Ostrava, Czech Republic; 2 Faculty of Mechanical Engineering, Brno University of Technology, Technická 2896, 616 00 Brno, Czech Republic; 3 Institute of Physics of Materials, Czech Academy of Science, Žižkova 22, 616 00 Brno, Czech Republic

**Keywords:** electro slag remelting, slag, non-metallic inclusion, chemical composition, thermodynamics

## Abstract

The purity of a steel is an important factor influencing the quality of the final products. Therefore, it is important to optimize the existing and develop new steelmaking technologies that affect the resulting purity. Electro slag remelting is a technology of tertiary metallurgy, which can advantageously be used to fabricate high quality steels. The study presents selected theoretical aspects of oxide systems and their specific influences on effectiveness of the electro slag remelting technology. The aim of this work was to experimentally analyze the purity of a tool steel fabricated by electro slag remelting using two different oxide systems (fused slags). The core of the study is the determination of the overall presence of elements in the steels, a thorough investigation of the presence of (not only) oxide-based inclusions within the investigated tool steel, and a detailed analysis of their chemical composition, including the size of these non-metallic inclusions, using energy dispersive X-ray (EDX) on the scanning electron microscope (SEM). Last but not least, the determination of the modification of the occurring non-metallic inclusions and verification of the experimentally acquired results as well as the calculation of the liquid and solid temperature and the calculation of the viscosity of the slags using the FactSage calculation software was performed. The results showed that the used slag influenced especially the occurrence of Mg and Al-based oxide inclusions. The CaS-type inclusions were present within all of the examined samples. The slag type influenced not only the typical morphology and size of the inclusions (especially of the CaS type), but also the tendency of the steel to exhibit localized corrosion when exposed to the ambient environment. This research can contribute to a better understanding of the effect of oxidation systems on the resulting purity and properties of ESR steels, thereby advancing the production of tool steels with higher quality and performance requirements.

## 1. Introduction

The ever increasing demands on the lifetime and performance of metallic materials go hand in hand with the research and development of innovative and improved production technologies [1,2,3]. Even in the contemporary world, steels are highly demanded and used not only for (massive) constructions [4,5], but also for various applications and components in the transportation [6,7], power engineering [8,9,10], or medicine [11,12]. Steels are typically cast and then processed into products featuring final shapes by methods of plastic deformation (conventional, e.g., [13,14], as well as unconventional, e.g., [15,16,17], or their combinations [18,19]). Nevertheless, the modern fabrication methods include also powder metallurgy [20,21,22] and additive manufacturing (although such methods are suitable for relatively smaller components and still require post-processing to eliminate defects) [23,24]. The ways to increase the performance of steels include not only performing controlled thermomechanical forming treatments, e.g., with optimized heating used to control the microstructures [25,26,27], but also improving the purity of steels during casting [28,29,30], which improves their performance from the very beginning of processing. Among the methods of increasing the purity of steels during casting is, for example, electro slag remelting (ESR) [31,32,33].

The principle of ESR is to melt a steel electrode through a layer of slag in a water-cooled copper mold, where the steel gradually solidifies to form an ingot [34,35,36]. The directed solidification of the ingot also creates favorable conditions for the excluding of inclusions and the release of gases [37,38,39]. ESR processing has a positive effect on improving the purity and mechanical and technological properties of steels. It is used for the technically and economically justified production of selected grades and brands of special alloy steels [40,41,42]. ESR significantly improves both the macrostructure and microstructure of the steel. Directed crystallization results in a uniform structure without internal defects [43,44,45]. The high micro-purity of the remelted steel has the influence of reducing the anisotropy of mechanical and physical properties [46], while increasing its formability. The steel can thus further be processed even by challenging methods (e.g., Severe Plastic Deformation, SPD [47,48,49]). The efficiency of ESR is depends on the chemical composition and physical properties of the slags used, their electrical conductivity, and appropriate viscosity [50,51,52]. During ESR, it is usually also desirable to achieve the highest possible degree of desulphurization [53] or dephosphorization. To obtain an ESR steel with a minimum possible content of non-metallic inclusions, it is necessary to select a slag capable of absorbing and dissolving these inclusions [54,55,56].

Schneider et al. [57] investigated the effects of different Al_2_O_3_ contents ranging from 0 to 33 wt. % using slags with chemical composition of Al_2_O_3_, SiO_2_, and MgO during steel remelting, focusing on changes in the chemical composition related to changes in type, content, distribution, size, and composition of non-metallic inclusions. The Al_2_O_3_ content in the slag has a significant effect on the Al, O, and S contents in the steel after remelting, and the amount of non-metallic inclusions occurring after remelting is strongly dependent on the concentration of Al_2_O_3_ in the slag. Lower Al_2_O_3_ contents resulted in lower elements concentrations in the ingots. Shi et al. [58] characterized the effects of the reoxidation of liquid steel during remelting and different SiO_2_ contents in the slag on the chemical compositions of oxide inclusions and the contents of alloying elements (Si and Al) in the steel. The composition of the slags used for the experiments was CaF_2_, CaO, Al_2_O_3_, MgO, and SiO_2_. The concentration of Al in the liquid steel pool was significantly reduced and accompanied by a decrease in Si losses with increasing SiO_2_ content in the slag. The O content in the steel increased significantly due to the reoxidation of the liquid steel pool during remelting. Schneider et al. [59] further studied the influence of different slag compositions, CaF_2_ content ranging from 0 to 60 wt. %, and corresponding to a wide range of electrical conductivity on slag movement, surface temperature, and thickness, as well as their influence on the occurring chemical reactions and removal of non-metallic inclusions. The used slags containing CaF_2_, CaO, Al_2_O_3_, SiO_2_, and MgO tended to reduce the content of larger non-metallic inclusions, especially sulfides, and increase the number of fine oxides during remelting. Wang et al. [60] studied the evolution of inclusions in remelted electrodes with different Ca contents refined by the ESR process. The chemical composition of the ESR slag was 60 wt. % of CaF_2_, 20 wt. % of CaO, and 20 wt. % of Al_2_O_3_. They analyzed the formation and development of sulfide and oxide inclusions. The inclusions in the steel after remelting were of Al_2_O_3_, CaO-Al_2_O_3_, and CaO-MgO-Al_2_O_3_ types. In contrast, MnS and CrS inclusions were completely removed from the steel. The different Ca contents in the electrodes had no effect on the size distribution of the inclusions in the remelted ingots. Wang et al. [61] investigated the evolution of non-metallic inclusions in austenitic refractory steel with different Ce contents during remelting. The pre-melted slag containing of CaF_2_, CaO, MgO, Al_2_O_3_, SiO_2_, and FeO. All the inclusions in the initial Ce-free remelted electrode were MgO-Al_2_O_3_-based, and some of these inclusions were removed from the steel during remelting by absorption of the molten slag. The Ce-added inclusions were present in the Ce_2_O_3_, CeAlO_3_, and Ce_2_O_2_S-based steels when the initial Ce content of the electrode was in the range of 0.016–0.300 wt. %. Shi et al. [62] studied the mechanism of formation of non-metallic inclusions and modification of the MgO·Al_2_O_3_ spinel by addition of Ca or Al during remelting of the H13 die steel. The spinel-type inclusions in the electrode were mainly modified to CaO-MgO-Al_2_O_3_, and some to the CaO-Al_2_O_3_-type inclusions. In the case of modification by Al, all the oxide inclusions present in the steel after remelting were of the MgO·Al_2_O_3_-spinel type. It was used in the pre-melted slag (60 wt. % of CaF_2_, 20 wt. % of CaO and 20 wt. % of Al_2_O_3_), which was roasted at 773 K (500 °C). Last but not least, Wang et al. [63] used numerical simulations to analyze the movement of non-metallic inclusions as a function of the electrical energy applied during the remelting of the steel to ESR. For the simulations, a slag composition of 75 wt. % of CaF_2_ and 25 wt. % of Al_2_O_3_ was used. The slag cap thickness remained constant at 60 mm. The simulations showed that the removal of non-metallic inclusions featuring diameters of 1–10 µm increased with the increasing electric current. Inclusions with diameters of 3–5 µm were removed in the range of currents of 1000–1600 A, but the removal of such inclusions was reduced up to the current of 1800 A. In addition to the size of the non-metallic inclusions, the density of the inclusions also influences their removal during ESR of a steel.

The results of the above studies show that the design and optimization of the slag regime during production, as well as study of the microstructure and chemical composition of the remelted steels, are of a high importance to produce steels with enhanced performances. Therefore, the primary objective of this study was to analyze the chemical compositions and microstructures of the investigated steel in detail, and the focus was especially on the occurrence of non-metallic inclusions in relation with the used oxide system (i.e., used ESR slag). The originality of the herein presented research is primarily in the fact that the used steel and slags have the exact chemical compositions as real commercially available products fabricated by European companies, such ISOMAG GmbH, Austria (slag producer), and ŽĎAS a.s., Czech Republic (steel producer), and thus the acquired results are directly applicable industrially. The study was supplemented with an analysis of the thermophysical properties of these oxide systems.

## 2. Results and Discussion

### 2.1. Energy Dispersive X-Ray Analysis of the Chemical Compositions

**Figure 1:**
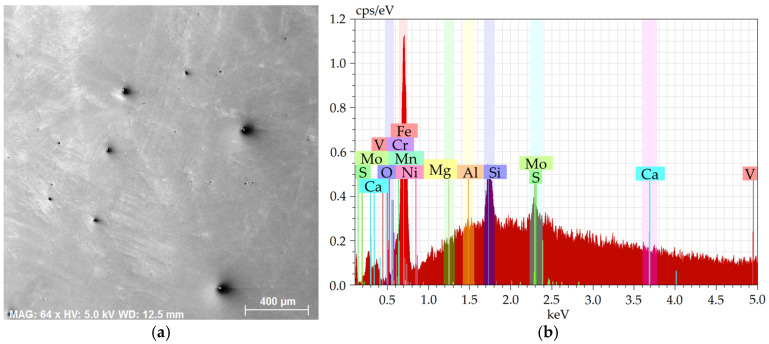
Sample 601: (**a**) scanned area; (**b**) chemical composition.

**Figure 2:**
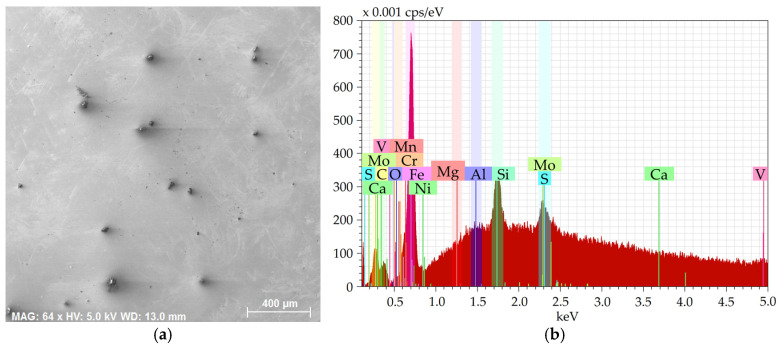
Sample 602: (**a**) scanned area; (**b**) chemical composition.

**Figure 3:**
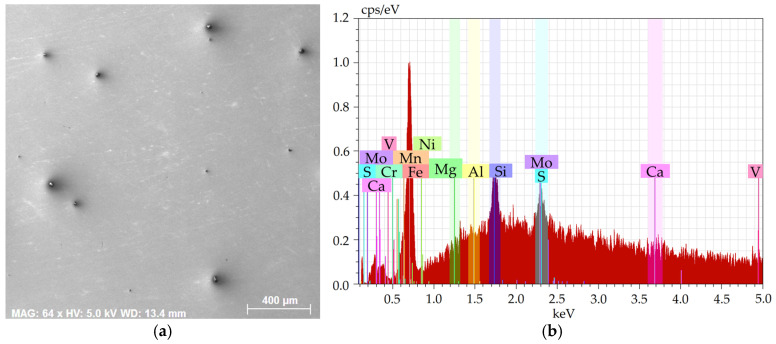
Sample 276: (**a**) scanned area; (**b**) chemical composition.

**Figure 4:**
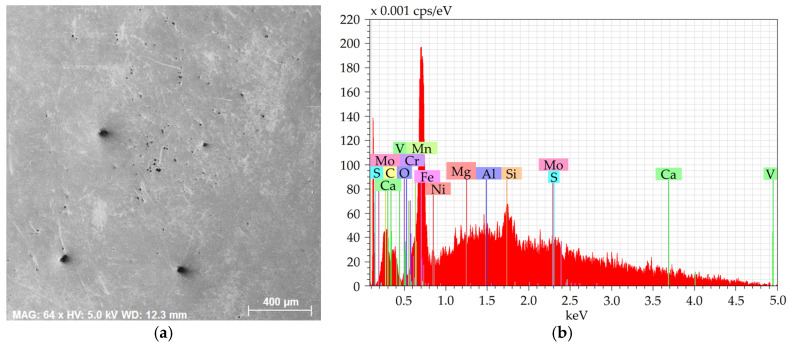
Sample 277: (**a**) scanned area; (**b**) chemical composition.

Figure 1a shows an SEM-SE image of the investigated 2 × 2 mm^2^ area from the 601 sample, while Figure 1b depicts the EDX chart from the respective area. Figure 2a then shows an SEM-SE image of the 2 × 2 mm^2^ area from the 602 sample, and Figure 2b depicts the EDX chart from the respective area. Figure 3a shows an SEM-SE image of the investigated area from the 276 sample, while Figure 3b depicts the EDX chart from the respective area. Lastly, Figure 4a shows an SEM-SE image of the investigated area from the 277 sample, and Figure 4b depicts the EDX chart from the respective area.

The charts depict that the samples contained the majority of Fe plus other elements, especially Si, Mg, Al, Ca, and also C, S, and O. The majority of the Mg, Al, Ca, and S were bound within more or less coarse inclusions, which will further be characterized for each examined steel batch.

**Figure 5:**
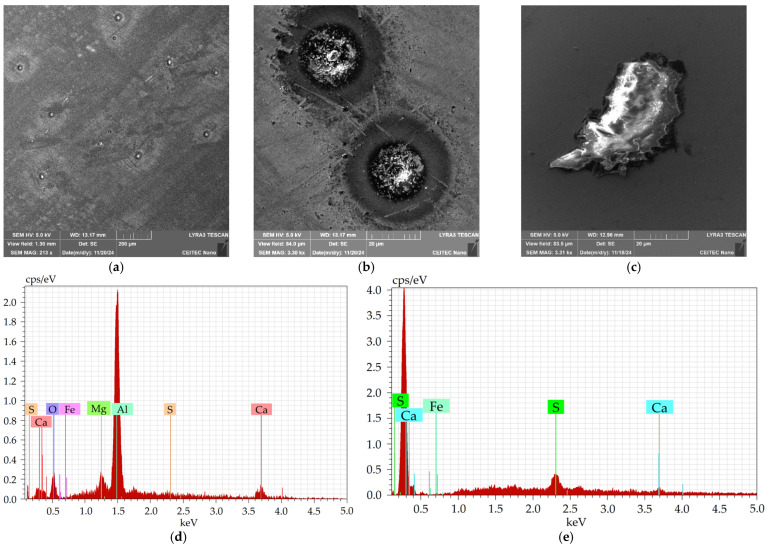
Sample 601: (**a**) SEM-SE images: general overview; (**b**) Al_2_O_3_-MgO-type inclusions; (**c**) CaS type-inclusion. EDX chart of chemical composition of (**d**) Al_2_O_3_-MgO-type inclusion; and (**e**) CaS-type inclusion.

Figure 5a shows an SEM-SE image depicting examples of the coarsest inclusions present within the 601 sample. The inclusions were of two types, a more or less spherical Al_2_O_3_-MgO type (depicted in greater detail in Figure 5b), and a sharp-edged CaS type (depicted in greater detail in Figure 5c). Typical chemical compositions of the inclusions are depicted in Table 1 (spherical inclusions) and Table 2 (sharp-edged inclusions), as well as in the EDX graphs in Figure 5d (spherical inclusions) and Figure 5e (sharp-edged inclusions); note that the presence of Fe was detected from the surroundings. Table 3 then shows the size distribution of the spherical inclusions, whereas Table 4 depicts the size distribution of the sharp-edged inclusions. As documented by the tables, the majority of the Al_2_O_3_-MgO-type inclusions had the diameters around 20 µm, and the average size of such a spherical inclusion was 20.7 µm. On the other hand, the majority of the sharp-edged CaS-type inclusions had diameters between 20 and 50 µm, and the average size of such an inclusion was 41.9 µm.

**Figure 6:**
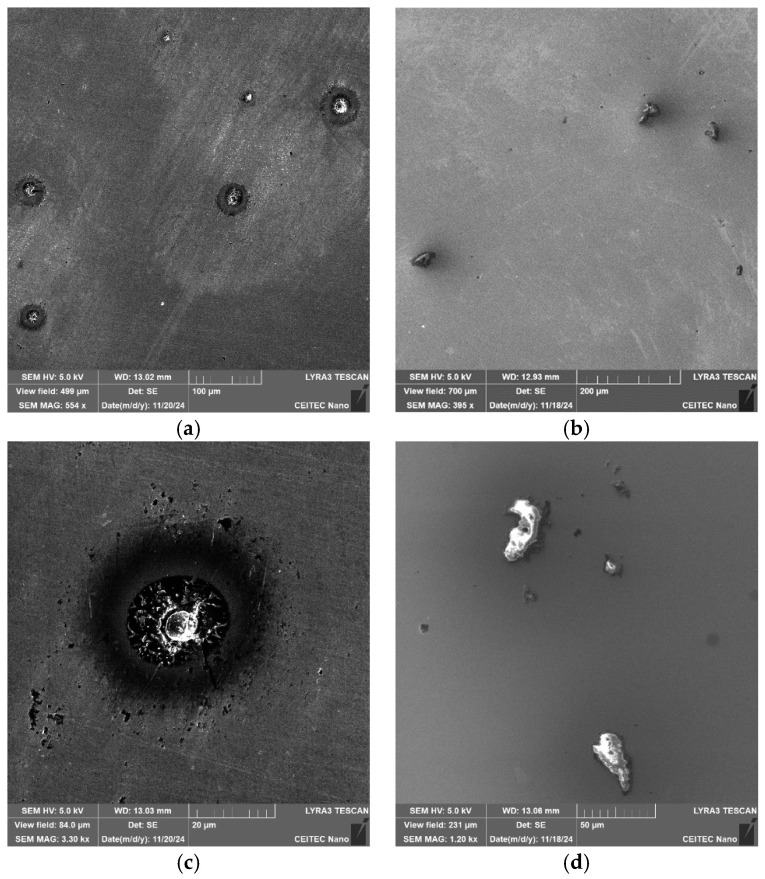
Sample 602: (**a**,**b**) SEM-SE images; (**c**) Al_2_O_3_-MgO-type inclusions; and (**d**) CaS-type inclusion.

Figure 6a,b show SEM-SE images of the coarsest inclusions present within the 602 sample. Similarly to the 601 sample, the inclusions within the 602 one, were of the Al_2_O_3_-MgO and CaS types. Figure 6c,d show detailed SEM-SE images of the Al_2_O_3_-MgO- and CaS-type inclusions, respectively. As can be seen from the figures, the Al_2_O_3_-MgO-type inclusions were of more or less spherical shapes, while the CaS inclusions were sharp-edged, similar to the 601 sample. Table 5 shows the size distribution of the spherical inclusions, while Table 6 depicts the size distribution of the sharp-edged inclusions. The data in the tables reveals that the average size of the precipitates within the 602 sample was slightly smaller than within the 601 one, especially with regards to the CaS-type inclusions. The average size of the spherical inclusions was 19.2 µm, which is comparable to the average size of such inclusions within the 601 sample. On the other hand, the average size of the sharp-edged inclusions was 32.3 µm, which is almost by 25% smaller than for the 601 sample.

**Figure 7:**
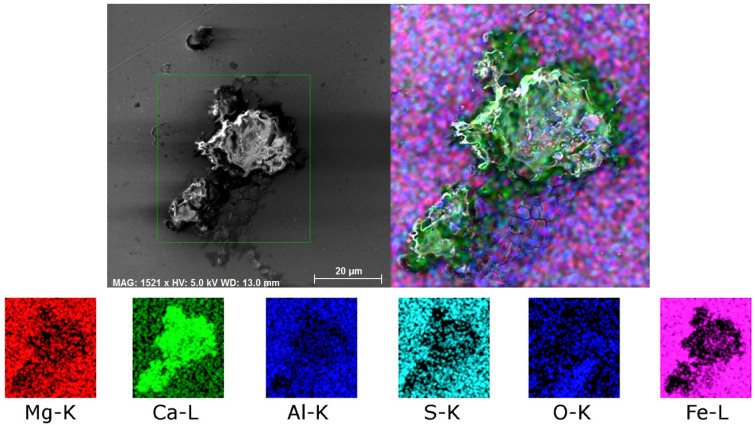
Distribution of elements within a crust-like inclusion in 602 sample.

Among the two typical types of inclusions, the 602 sample also featured crust-like inclusions. Figure 7 shows a detailed map of chemical composition of a crust-like inclusion present within the 602 sample. As can be seen, such an inclusion primarily consisted of Ca, other elements (Mg, Al) were present, but with negligible contrasts. These were CaS and CaS-CaO inclusions.

**Figure 8:**
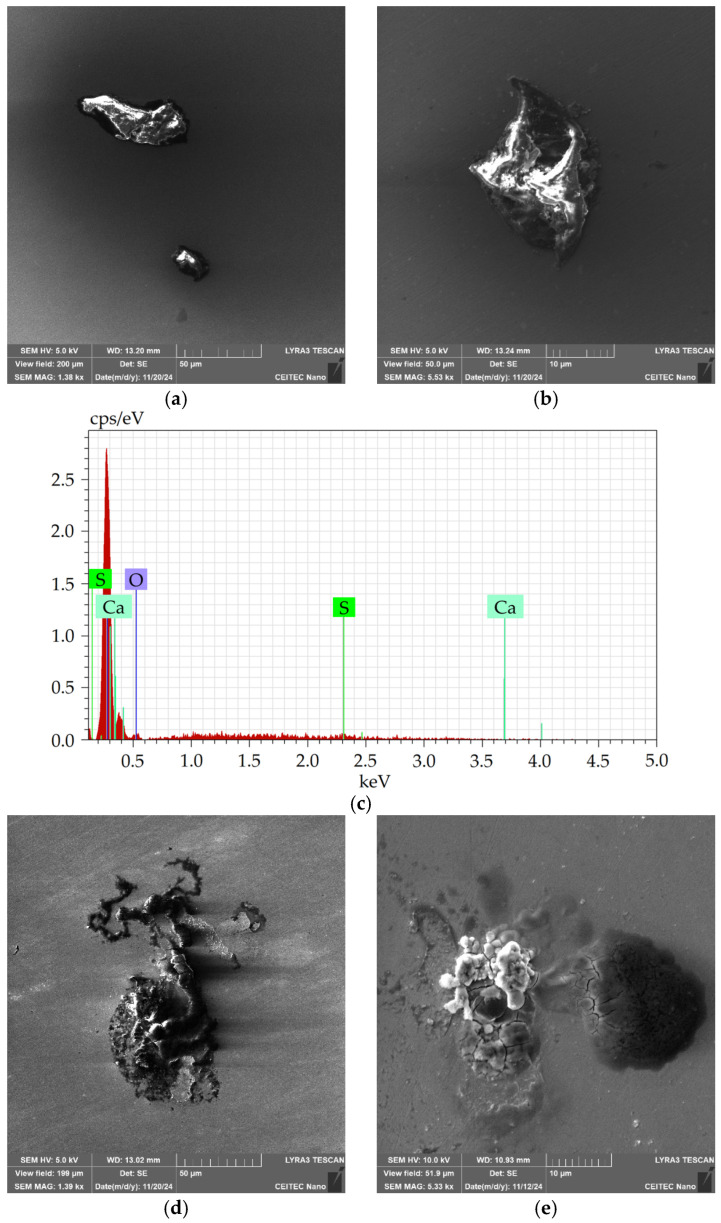
Sample 276: (**a**,**b**) SEM-SE images: CaS-type inclusions; (**c**) EDX chart of chemical composition of CaS-type inclusion; and (**d**,**e**) SEM-SE images of crust-like inclusions.

The 276 sample also featured the presence of coarse sharp-edged inclusions, see Figure 8a,b. However, this sample did not exhibit any notable presence of the spherical Al_2_O_3_-MgO-type inclusions. The coarse inclusions were of the CaS type, see the EDX graph in Figure 8c showing a typical chemical composition of such an inclusion within the 276 sample. Among the sharp-edged inclusions, the 276 sample also exhibited crust-like ones, which points to a certain similarity with the 602 sample. However, the inclusion within the 276 sample were of a slightly different nature, see Figure 8d,e (and compare to Figure 7). Table 7 then depicts the size distribution of the sharp-edged CaS-type inclusions. The size distribution evidently featured greater variations than that observed for the 601 and 602 samples, although the average inclusion size was comparable to that acquired for the 601 sample). The 276 sample featured a mixture of finer and very coarse inclusions, with the average size of 42.6 µm.

**Figure 9:**
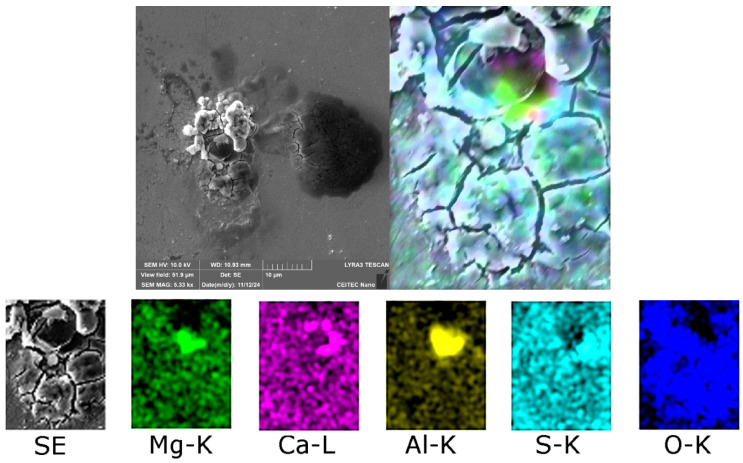
Distribution of elements within crust-like inclusion in 276 sample.

Figure 9 depicts detailed maps depicting the chemical composition of a crust-like inclusion present within the 276 sample (see Figure 8e). As can be seen, such an inclusion consisted of a mixture of Ca, Mg, and Al (complex non-metallic inclusions of Al_2_O_3_-MgO-CaO) oxides; the crust-like precipitates also typically featured the presence of Al and Mg oxides (complex non-metallic inclusions of Al_2_O_3_-MgO), as clearly seen in the maps in Figure 9. The crust featured an increase in the presence of S, too.

**Figure 10:**
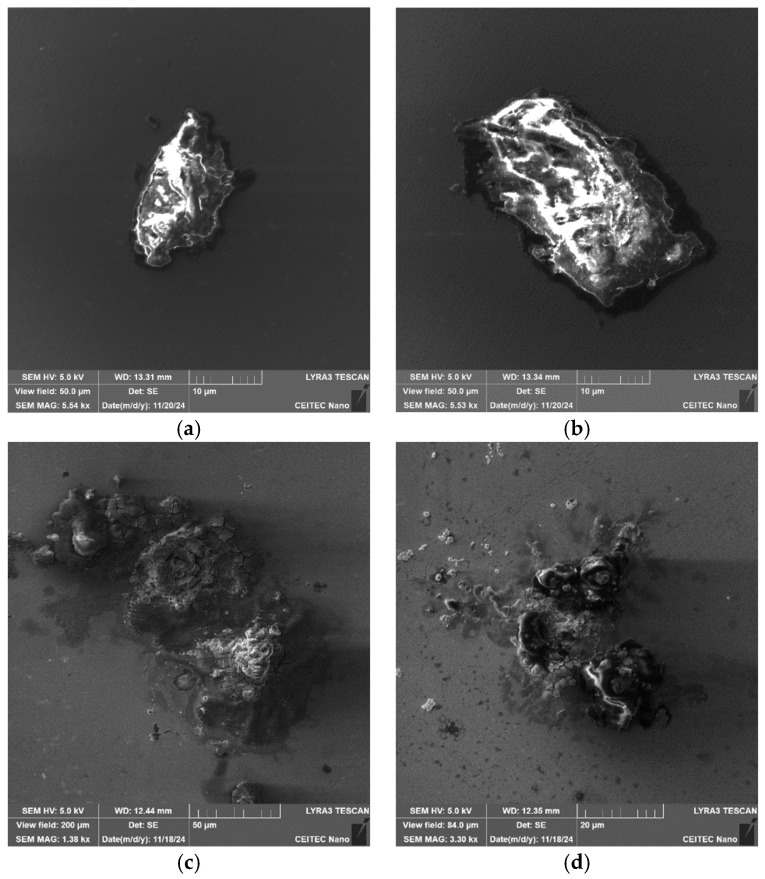
Sample 277: (**a**,**b**) SEM-SE images: CaS-type inclusions; and (**c**,**d**) crust-like inclusions.

Last but not least, the analysis performed for the 277 sample revealed that this sample exhibited the presence of coarse sharp-edged CaS inclusions, similar to the other examined samples (see Figure 10a,b). However, compared to the other samples, the presence of such inclusions was scarce; the majority of the inclusions present within the 277 sample were the crust-like ones, as seen in Figure 10c,d. Table 8 depicts the size distribution of the (scarcely present) sharp-edged inclusions. As can be seen, the size variation featured a lower range when compared to the 276 sample. Also, the average size of an inclusion was smaller within the 277 sample than within the 276 one. Nevertheless, the average size was comparable to that calculated for the 602 sample. An intriguing fact is that the average size of a CaS-type inclusion was comparable for samples 601 and 276, and 602 and 276.

**Figure 11:**
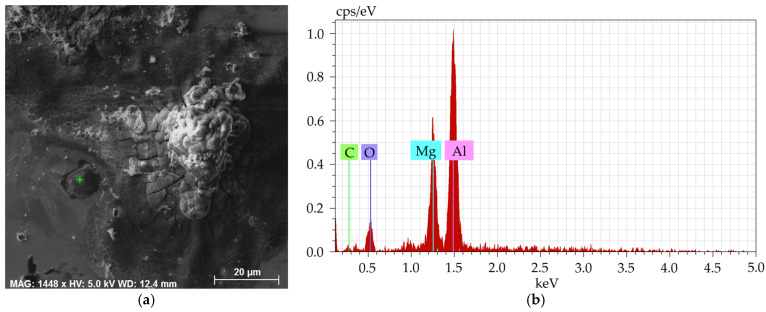
Sample 277: (**a**) SEM-SE image of crust-like inclusion containing non-formable Al_2_O_3_-MgO; (**b**) respective chemical composition.

**Figure 12:**
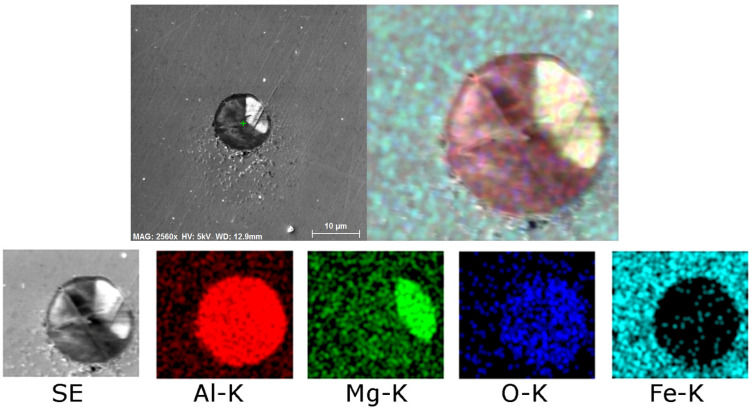
Distribution of elements within oxidic inclusion in 277 sample.

Detailed depiction of the presence of non-formable oxidic inclusions, occurring typically as a part of the crust-like inclusions, is shown in Figure 11a, depicting a detailed SEM-SE image of the oxide inclusion with marked location of analysis of chemical composition, the results of which are then shown in Figure 11b. Figure 12 then shows detailed maps of chemical composition of an oxidic inclusion present within the 277 sample. As can be seen, similar to the 276 sample, the chemical composition of such an inclusion was a mixture of oxides of Ca, Mg, and Al (complex non-metallic inclusions of Al_2_O_3_-MgO-CaO), the crust featured an increased presence of S, as well as the presence of Al and Mg oxides (complex non-metallic inclusions of Al_2_O_3_-MgO), as clearly seen in the maps in Figure 12. Finally, Table 9 depicts a typical chemical composition of such an oxidic inclusion.

Overall, all the examined samples exhibited the presence of Al_2_O_3_-MgO-type spherical inclusions and CaS-type sharp-edged inclusions. The average size of the sharp-edged CaS-type inclusions was larger for the 601 sample than for the 602 one, and for the 276 sample than for the 277 one. Nevertheless, the average size of the CaS inclusions within the 601 sample was comparable to that acquired for the 276 sample, and the average size acquired for the 602 sample was comparable to that acquired for the 277 one. Moreover, among the CaS-type inclusions, the 601 and 602 samples featured a frequent presence of spherical Al_2_O_3_-MgO-type inclusions, contrary to the 276 and 277 samples, which featured crust-like inclusions with a local presence of coarse Al_2_O_3_-MgO inclusions. The occurrence of the Al_2_O_3_-MgO-type inclusions was thus more frequent within the 601 and 602 samples.

**Figure 13:**
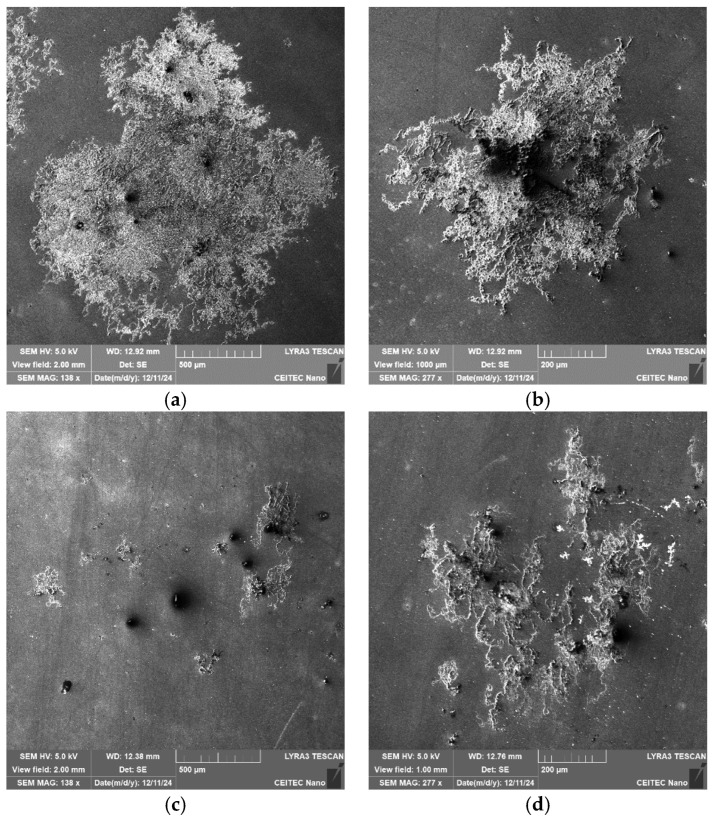
Surface corrosion after two-month exposure to regular atmosphere: (**a**,**b**) sample 276; (**c**,**d**) sample 277.

Another intriguing fact is that the 276 and 277 samples are also highly prone to atmospheric corrosion. After two months of exposure of the polished samples to the atmosphere, the 276 and 277 samples started to exhibit traces of localized corrosion (contrary to the 601 and 602 samples, which did not exhibit any corrosion during that time period). Figure 13a,b show SEM-SE images depicting the corrosion products on the surface of the 276 sample, while Figure 13c,d show SEM-SE images of the corrosion products on the surface of the 277 sample. The corrosion evidently started to nucleate around the inclusions and was more developed on the 276 sample than on the 277 one.

### 2.2. Verification of EDX Analysis Using Factsage

As regards the chemical composition of non-metallic inclusions in the tool steel, the amount of MgO-based non-metallic inclusions increased significantly after the ESR technology. The Equilib module of the FactSage software was used to verify the results acquired experimentally via the EDX analysis, i.e., to confirm/refute the hypothesis whether these inclusions occurred as a result of the concentration of MgO in the slag. The chemical composition of the tool steel and the chemical composition of the ESR slags were used to calculate changes in the chemical compositions of the non-metallic inclusions. As an example, the calculation of the modification of the non-metallic inclusions using the AKF 235 slag, which was characterized by a relatively high MgO content (3 wt. %), is further presented.

**Figure 14:**
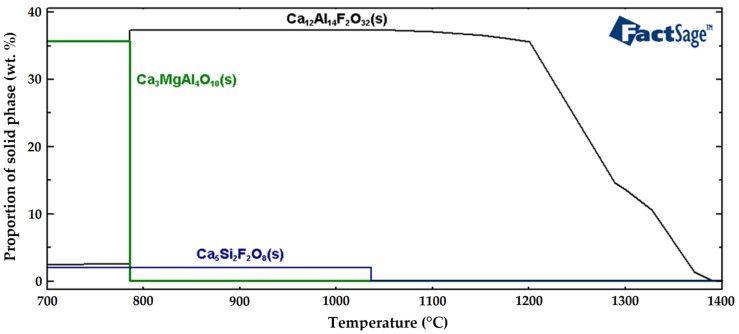
Solid phase formation in case of AKF 235 slag.

Figure 14 shows the resulting graphical representation of the solid non-metallic phases formed in the tool steel during solidification. The formation of the Ca_12_Al_14_F_2_O_32_(s) solid phase started at 1370 °C. The wt. % of the Ca_12_Al_14_F_2_O_32_(s) solid phase then decreased from 37 wt. % to 2.5 wt. % at the solidification temperature of 786 °C, and then remained constant when further cooled down to 20 °C. The formation of the Ca_5_Si_2_F_2_O_8_(s) solid phase started at 1036 °C and was constant throughout the entire range of examined temperatures, down to 20 °C, with the maximum solid phase fraction of 2 wt. %. The formation of the Ca_3_MgA_4_O_10_(s) solid phase occurred at 786 °C. At this temperature, its concentration increased dramatically, to 36 wt. %. The results of changing of the chemical compositions of non-metallic inclusions calculated using the FactSage software confirmed that when MgO is present in the slag, the majority of the non-metallic inclusions in the tool steel consists of complex MgO-based inclusions. Calculations performed using the FactSage software also indicated the presence of Ca-based phases, which is also consistent with the EDX analyses of the examined samples. Especially in the cases of the 276 and 277 samples (remelted with the AKF 235 slag), the chemical compositions of the complex non-metallic inclusions showed mixtures of Ca, Mg, and Al oxides.

**Figure 15:**
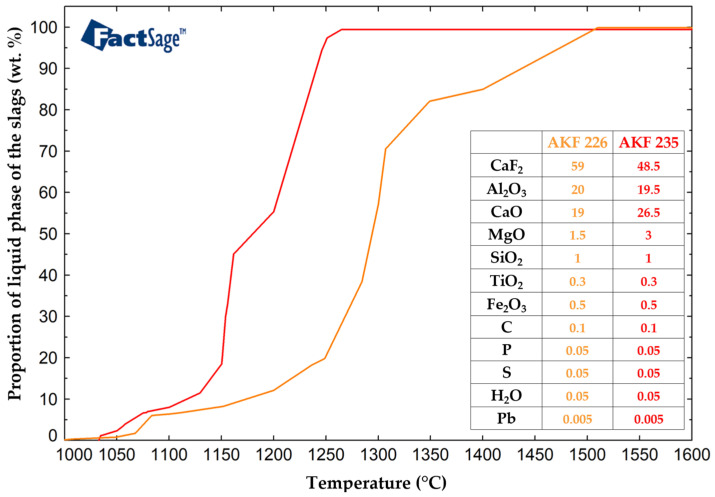
Solid and liquid temperatures for ESR AKF 226 and AKF 235 slags.

In addition to the characterization of non-metallic inclusions, other important parameters of the investigated slags (oxide systems) [64,65], which influence the removal of non-metallic inclusions and thus the purity of the remelted steel, were also calculated using FactSage. The ESR melting intervals of the slags were determined in the same Equilib module. Important parameters in the ESR process were the temperature of the liquid slag and the temperature interval between the solid and liquid phases. The slag melting interval calculations were performed in the temperature range of 1000 °C to 1450 °C, with a step of 100 °C, and the equilibrium was set as normal + transitions. For the AKF 226 slag, the calculated solid and liquid temperatures were 1049 °C and 1510 °C, while for the AKF 235 slag, the calculated solid and liquid temperatures were 1033 °C and 1261 °C, respectively. As can be seen, there was a significant difference between the solid and liquid temperatures of the AKF 226 slag, i.e., 461 °C, which was more than double the value acquired for the AKF 235 slag (228 °C). The results of the calculations performed using the FactSage software to determine the liquid phase fraction of the studied slags are in Figure 15.

Last but not least, the dynamic viscosities of the slags as a function of temperature were calculated in the viscosity module of the FactSage software for all the components present in each slag. The calculations were performed in the working conditions range of the ESR slags, i.e., in the interval of 1750–1800 °C. Table 10 shows the calculated dynamic viscosity values. From the calculated values, it can be seen that the value of the dynamic viscosity decreased with increasing temperature for both the studied slags. At the same time, the dynamic viscosity of the AKF 226 slag decreased compared to the AKF 235 slag, mainly due to the higher CaF_2_ content in the slag. In addition to the effect of CaF_2_ on the viscosity of the slag, CaF_2_ has also a suitable fusibility and relatively high evaporation temperature, which allow the process to initiate relatively quickly and easily. It also features favorable desulphurization efficiency, and, overall, contributes to the stability of the ESR process. The effect of the temperature and viscosity of the slag is very important from the viewpoint of formation of the structure of an ingot, especially as regards its head. The release of gases from the last fraction of liquid steel crystallizing in the head of the ingot is facilitated by the use of easily fusible slags. The appearance of axial integrity in the head of the ingot is associated with the use of hardly fusible slags.

## 3. Materials and Methods

The study focused on a detailed analysis of tool steels remelted using ESR equipment. The typical average chemical composition of such a remelted tool steel is shown in Table 11. As slags are an important component in the processing of a steel using ESR, the effect of these oxide systems on the purity of the steel fabricated was investigated. Two slags with CaF_2_, Al_2_O_3_, CaO, and MgO as their main constituents were used for the study. These slags are commercially available, e.g., from ISOMAG GmbH, Kraubath an der Mur, Austria, and their chemical compositions are shown in Table 12.

A total of four forgings of tool steels remelted by the ESR technology were analyzed, with several samples taken and analyzed from each forging. Samples marked 601 and 602 (remelting 1 and 2) are from forgings remelted with the AKF 226 slag, while samples marked 276 and 277 (remelting 3 and 4) are from forgings remelted with the AKF 235 slag.

The chemical compositions of the examined steels, including the compositions and morphologies of the occurring inclusions, were examined using the Energy Dispersive X-ray Spectroscopy (EDX) of the Focused Ion Beam/scanning electron microscope TESCAN LYRA3 microscope (Tescan Brno, s.r.o., Brno, Czech Republic) at the CEITEC Nano Research Infrastructure, Brno. In order to reliably examine the presence of light elements of interest, such as Mg, Al, or Ca, the analyses were performed at a low accelerating voltage of 5 keV. To assess the overall presence of the elements within the microstructures of the prepared steels, an EDX analysis from a relatively large area of 2 × 2 mm^2^ was performed for each sample at first. Detailed EDX analyses were then performed to examine the individual inclusions, including their chemical compositions. Last but not least, the sizes of the inclusions were also measured; the size of an inclusion was measured as the largest distance between two points (locations) within the inclusion.

In order to verify the experimental evaluation, i.e., confirm the analysis of samples of tool steel forgings processed by the ESR technology (as shown in Table 11), thermodynamic calculations for determination of the modification of the occurring non-metallic inclusions and their verification were performed using FactSage software (version 8.2 by GTT-Technologies, Herzogenrath, Germany). The FactSage software is one of the largest fully integrated database computer systems in chemical thermodynamics. It is also suitable for determination of important metallurgical parameters based on chemical composition and temperature [66,67,68,69,70]. To calculate the modification of non-metallic inclusions in the tool steel, the chemical composition of the steel (see Table 11) was imported together with the calculated Fe content in the steel (sum of elements 100%). The average chemical composition of the slag (AKF 226 and AKF 235) was imported together with the chemical composition of the steel. Calculations of the modification of the non-metallic inclusions were performed in the temperature range from 300 °C to 1400 °C, with steps of 100 °C, and the equilibrium was set as normal + transitions.

## 4. Conclusions

The focus of the presented study was especially on the detailed analysis of the occurrence and chemical compositions of oxide-based inclusions within the investigated tool steel, in relation with the type of slag used during the electro slag remelting production process. The determination of the thermophysical properties of the studied oxide systems and verification of the experimentally acquired results were performed using FactSage calculation software. The main acquired results are as follows:

- Both the studied oxide systems affected formation of non-metallic inclusions of Al_2_O_3_-MgO and CaS type in all the analyzed tool steels.
- The Al_2_O_3_-MgO-based inclusions occurred mainly as a result of MgO content in the slag, while the amount of MgO in the slag was not found to be essential for the amount and size of these inclusions in the studied steel after ESR.
- In all cases, there were no significant size differences between the complex Al_2_O_3_-MgO-based non-metallic inclusions due to their modification.
- The size range of spherical Al_2_O_3_-MgO-type non-metallic inclusions was much smaller than that of the sharp-edged CaS-type non-metallic inclusions.
- Calculations using the FactSage software confirmed the presence of complex non-metallic inclusions in the tool steel, and the significant increase in the Ca-Mg-Al-O-based solid phase confirmed the presence of these types of inclusions in the steel after ESR.

## Figures and Tables

**Table 1:** Chemical composition of a typical spherical inclusion within the 601 sample (at. %).

Element	Al	Mg	O
Content in at. %	49.3	38.8	11.9

**Table 2:** Chemical composition of a typical sharp-edged inclusion within the 601 sample (at. %).

Element	Fe	Ca	S
Content in at. %	56.1	33.5	10.4

**Table 3:** Sizes of Al_2_O_3_-MgO precipitates within the 601 sample.

Precipitate No.	1	2	3	4	5	6	7	8	9	10	AVG.
Largest dimension (µm)	18	20	19	24	23	16	19	20	24	24	20.7

**Table 4:** Sizes of CaS precipitates within the 601 sample.

Precipitate No.	1	2	3	4	5	6	7	8	9	10	AVG.
Largest dimension (µm)	58	22	20	42	68	26	33	62	49	39	41.9

**Table 5:** Sizes of Al_2_O_3_-MgO precipitates within the 602 sample.

Precipitate No.	1	2	3	4	5	6	7	8	9	10	AVG.
Largest dimension (µm)	13	11	23	28	22	16	19	18	19	23	19.2

**Table 6:** Sizes of CaS precipitates within the 602 sample.

Precipitate No.	1	2	3	4	5	6	7	8	9	10	AVG.
Largest dimension (µm)	22	16	29	39	44	42	38	22	38	33	32.3

**Table 7:** Sizes of CaS precipitates within the 276 sample.

Precipitate No.	1	2	3	4	5	6	7	8	9	10	AVG.
Largest dimension (µm)	60	26	24	82	26	32	36	34	78	28	42.6

**Table 8:** Sizes of CaS precipitates within the 277 sample.

Precipitate No.	1	2	3	4	5	6	7	8	9	10	AVG.
Largest dimension (µm)	22	16	39	42	19	26	27	44	49	19	30.3

**Table 9:** Chemical composition of a typical Al_2_O_3_-MgO inclusion within the 277 sample (as depicted in Figure 11a) (at. %).

Element	Al	Mg	O
Content in at. %	54.9	34.2	10.9

**Table 10:** Results of calculations of dynamic viscosity of ESR AKF 226 and AKF 235 slags.

Slag	AKF 226		AKF 235	
Temperature (°C)	1750	1800	1750	1800
Viscosity (Pa·s)	0.017	0.015	0.013	0.011

**Table 11:** Chemical composition of used tool steel (wt. %).

C	Mn	Si	P	S	Cr	Ni	Mo	V	Al	Nb	Fe
0.39	0.40	0.97	0.025	0.003	5.08	0.40	1.20	0.43	0.02	0.03	Bal.

**Table 12:** Chemical compositions of commercially available ESR slags used for remelting (wt. %).

**AKF 226**											
CaF_2_	Al_2_O_3_	CaO	MgO	SiO_2_	TiO_2_	Fe_2_O_3_	C	P	S	H_2_O	Pb
59	20	19	1.5	1	0.3	0.5	0.1	0.05	0.05	0.05	0.005
**AKF 235**											
CaF_2_	Al_2_O_3_	CaO	MgO	SiO_2_	TiO_2_	Fe_2_O_3_	C	P	S	H_2_O	Pb
48.5	19.5	26.5	3	1	0.3	0.5	0.1	0.05	0.05	0.05	0.005

## Data Availability

The raw/processed data required to reproduce these findings cannot be shared at this time due to technical limitations.
